# Non-reversible and Reversible Heat Tolerance Plasticity in Tropical Intertidal Animals: Responding to Habitat Temperature Heterogeneity

**DOI:** 10.3389/fphys.2018.01909

**Published:** 2019-01-14

**Authors:** Amalina Brahim, Nurshahida Mustapha, David J. Marshall

**Affiliations:** Environmental and Life Sciences, Faculty of Science, Universiti Brunei Darussalam, Bandar Seri Begawan, Brunei

**Keywords:** developmental plasticity, *Echinolittorina malaccana*, habitat heterogeneity, heat resistance, thermal acclimation

## Abstract

The theory for thermal plasticity of tropical ectotherms has centered on terrestrial and open-water marine animals which experience reduced variation in diurnal and seasonal temperatures, conditions constraining plasticity selection. Tropical marine intertidal animals, however, experience complex habitat thermal heterogeneity, circumstances encouraging thermal plasticity selection. Using the tropical rocky-intertidal gastropod, *Echinolittorina malaccana*, we investigated heat tolerance plasticity in terms of laboratory acclimation and natural acclimatization of populations from thermally-dissimilar nearby shorelines. Laboratory treatments yielded similar capacities of snails from either population to acclimate their lethal thermal limit (LT_50_ variation was ∼2°C). However, the populations differed in the temperature range over which acclimatory adjustments could be made; LT_50_ plasticity occurred over a higher temperature range in the warm-shore snails compared to the cool-shore snails, giving an overall acclimation capacity for the populations combined of 2.9°C. In addition to confirming significant heat tolerance plasticity in tropical intertidal animals, these findings reveal two plasticity forms, reversible (laboratory acclimation) and non-reversible (population or shoreline specific) plasticity. The plasticity forms should account for different spatiotemporal scales of the environmental temperature variation; reversible plasticity for daily and tidal variations in microhabitat temperature and non-reversible plasticity for lifelong, shoreline temperature conditions. Non-reversible heat tolerance plasticity, likely established after larvae settle on the shore, should be energetically beneficial in preventing heat shock protein overexpression, but also should facilitate widespread colonization of coasts that support thermally-diverse shorelines. This first demonstration of different plasticity forms in benthic intertidal animals supports the hypothesis that habitat heterogeneity (irrespective of latitude) drives thermal plasticity selection. It further suggests that studies not making reference to different spatial scales of thermal heterogeneity, nor seeking how these may drive different thermal plasticity forms, risk misinterpreting ectothermic responses to environmental warming.

## Introduction

Thermal plasticity enables ectothermic animals to modify their lifetime responses to environmental temperature. In the context of climate warming, a complex theory for this plasticity has emerged, which considers its energetic benefits, environmental drivers and evolutionary constraints (see the *beneficial acclimation hypothesis*, and hypotheses for thermal variability, predictability and latitudinal effects; Leroi et al., 1994; Kingsolver and Huey, 1998; Wilson and Franklin, 2002; Angilletta et al., 2006; Deere and Chown, 2006; Gunderson and Stillman, 2015). This theory has, however, been developed with taxonomic and ecological biases toward terrestrial insects, lizards, amphibians, and marine fishes (Huey et al., 1999; Mitchell et al., 2011; Overgaard et al., 2011; Kingsolver et al., 2013; Phillips et al., 2015), to the exclusion largely of animals inhabiting marine intertidal zones (but see Stillman, 2003; Somero, 2010). Marine intertidal circumstances are important as the theory may not always apply to them, despite its assumed generality. For example, studies investigating environmental temperature variation as the primary driver of thermal plasticity selection, commonly consider latitudinal and seasonal effects (Angilletta, 2009). These studies frequently conclude that temperate species, which typically experience greater thermal variation possess a greater capacity for thermal acclimation than tropically-distributed species, which experience relatively limited thermal variation (Gunderson and Stillman, 2015; Rohr et al., 2018). Although this may be true for most tropical animals and habitats, tropical marine intertidal habitats often promote extreme and variable temperature conditions, likely to drive thermal plasticity selection (Helmuth et al., 2006a,b; Marshall et al., 2010, 2018; Denny et al., 2011; Gedan et al., 2011).

**FIGURE 1:**
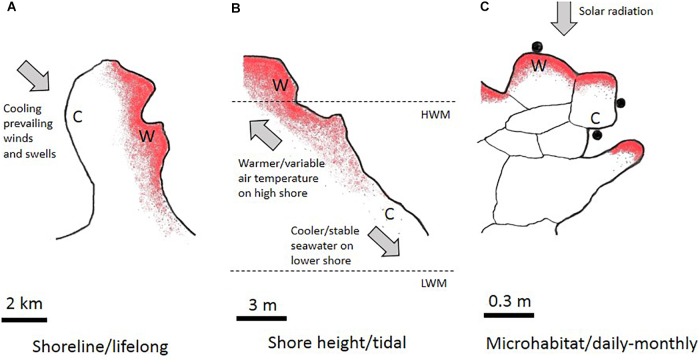
Concept for spatiotemporal scaling of rocky-shore thermal heterogeneity. **(A)** Aerial view of a coastal headland (2 km scale) showing the heat-load distribution (more intense red shading represents greater heat-load). One side of the headland (C) is exposed to prevailing winds and swells whereas the other side (W) is sheltered. Organisms experience either cooler or warmer shoreline conditions throughout their lifetimes. **(B)** Vertical profile of a rocky-shore (3 m scale), showing greater heat-loading in the higher shore (W) due to the variable air temperature. Relatively stable, cooler conditions (C) are experienced in the lower-shore. HWM, high water mark; LWM, low water mark. **(C)** Microhabitats of resting snails (0.3 m scale) showing the effect of solar heating on heat loads. Snails (solid circles) may retain these positions for months in the supratidal or may be redistributed after splashing during spring high-water or high monsoon swell conditions. Snails are primarily heated from the rock surface (see Marshall and Chua, 2012).

Thermal heterogeneity in benthic tropical intertidal ecosystems derives from multiple within- and between-shore effects (Figure 1). Within-shore temperature regimes depend primarily on the vertical position on the shore (low- to high-shore), which determines the degree of tidal inundation by relatively cool, thermally-stable seawater and concomitantly the period of exposure to warm, thermally-variable air. At any shore height, the microclimate at scales of below 1 m varies in relation to topography, slope, texture and color of the rocky substratum (Helmuth and Hofmann, 2001; Marshall et al., 2010; Denny et al., 2011; Gedan et al., 2011; Dong et al., 2017). Nearby shorelines which experience similar ambient air temperatures can, however, differ greatly in heat-loading in relation to aspect (north, south, east or west facing), slope, and level of protection from the prevailing winds and swells (Figure 1; Helmuth et al., 2006a,b).

From a temporal perspective, tropical rocky-shore thermal heterogeneity is encompassed within daily and tidal timeframes (Figure 1), rather than a seasonal timeframe as in temperate regions (Helmuth and Hofmann, 2001; Marshall et al., 2010; Denny et al., 2011; Gedan et al., 2011). Whereas conspecific individuals on the same shoreline often experience different thermal regimes in relation to different vertical or microhabitat distributions (see above), behavioral idiosyncrasies of some species result in the same individual undergoing rapid regime change within the narrow timeframe of a tidal cycle. This is exemplified by high-shore snails whose settling positions when the tide recedes are preferentially determined by desiccation risk avoidance rather than by thermal cues causing snails to rapidly stop crawling when rock surfaces become hot and dry (Monaco et al., 2017). New resting sites can be retained for weeks and can be much hotter (25 to >45°C in full sun-exposed individuals) or much cooler (25–35°C in individuals settling the shade) than the original sites occupied before tidal wetting and activity (Marshall et al., 2013). The exceptional habitat heterogeneity and dynamic variability of the thermal regimes experienced by many tropical intertidal animals should drive plasticity selection.

Because high-shore animals are particularly threatened by acute overheating (Williams and Morritt, 1995; Harley, 2008; Garrabou et al., 2009), heat tolerance plasticity should be under significant selection pressure, especially where behavioral thermoregulatory capabilities are limited. Although reversible plasticity is better known with respect to seasonality, this plasticity should also benefit intertidal animals facing variations in daily maximum temperatures. Superimposed on microhabitat thermal regimes are shoreline-specific thermal conditions, resulting in hotter microhabitats on hotter shores and *vice versa*, throughout an individual’s lifetime. Between-shore temperature differences present circumstances likely to drive non-reversible (or developmental) plasticity selection (Hoffmann et al., 2003; Angilletta, 2009; Seebacher et al., 2012, 2014; Beaman et al., 2016). Despite the important contributions of Somero and Stillman to ecological and evolutionary perspectives for intertidal thermal plasticity (Stillman, 2003; Somero, 2010), no previous studies have investigated different plasticity forms in benthic intertidal animals. Most of the research considering non-reversible thermal plasticity (developmental and transgenerational) concerns insects and fishes, and refers more commonly to performance than tolerance traits (Angilletta et al., 2006; Angilletta, 2009; Donelson et al., 2011, 2012; Beaman et al., 2016; Sørensen et al., 2016).

Gastropods represent a dominant ecological component of rocky intertidal zones, and nearly exclusively inhabit the uppermost shore level. Consequently, they have evolved complex behavioral and physiological mechanisms to endure energy gain constraints and resist extreme heat and desiccation exposures (Marshall and McQuaid, 2011; Marshall et al., 2011, 2013, 2015; Marshall and Chua, 2012; Verberk et al., 2016; Ng et al., 2017). The ubiquitous tropical high-shore gastropod, *Echinolittorina malaccana*, is emerging as a model species for exploring molecular heat stress innovations (Dong et al., 2011, 2017; Liao et al., 2017; Somero et al., 2017; Han et al., 2019). Contrary to the general theory predicting a trade-off between thermal acclimation capacity and basal heat tolerance, this thermophilic snail has been shown to exhibit substantial heat tolerance plasticity (Gunderson and Stillman, 2015; Marshall et al., 2018).

The present study aimed to determine whether the heat tolerance plasticity of *E. malaccana* snails could be described in terms of reversibility and non-reversibility. Reversible plasticity of the lethal temperature (LT_50_) was investigated from laboratory acclimation experiments. Non-reversible plasticity was assessed by comparing the thermal bands for lethal temperature acclimation of snail populations from warmer or cooler shorelines. Because reversible laboratory acclimation is expected to eliminate the effects of recent field temperature exposures, non-reversible acclimatization was assumed in cases where the thermal acclimation bands varied between the populations.

## Materials and Methods

### Snail Habitats and Thermal Regimes

*Echinolittorina malaccana* (Philippi 1847) occurs abundantly on rocky-shores throughout the Indo-Pacific (Reid, 2007). The local shores of Brunei Darussalam sustain two morphologically-distinct ecotypes, occupying different vertical zones. A brown ecotype inhabits the upper intertidal zone (roughly 1–2.5 m Chart Datum) and experiences tidal wetting, whereas a pale blue ecotype inhabits the supratidal zone (2.5- above 5 m Chart Datum) and is only wetted during high seas and monsoon swells. After periods of wetting and feeding, snails stop moving as the tide recedes, glue their shells to the rock surface, and withdraw into the shell (Marshall et al., 2011; Monaco et al., 2017). Because avoidance of desiccation while moving over hot dry rocks supersedes behavioral selection of thermally suitable resting (aestivating) sites, individuals often settle under direct sunlight (Marshall and Chua, 2012; Marshall et al., 2013; Monaco et al., 2017). Isolated resting snails undergo temperature-insensitive metabolic rate depression to overcome the energetic problem of high temperature exposure (Marshall and McQuaid, 2011; Marshall et al., 2011).

This study considered the intertidal brown ecotype. We determined the thermal regimes experienced at their upper distribution on a cool and a warm shoreline at Pantai Tungku, Brunei Darussalam (4.974°N, 114.867°E), between 21 June and 22 July 2018 (30 days during the warmest time of the year; Supplementary Figure S1). Pantai Tungku comprises a man-built promontory with artificial seawalls having diametrically-opposed orientations and carrying very different heat-loads. Study sites were established on the seawalls around 2 km apart, along the contour of the coast; the cool shoreline (CS) comprised a north-west-facing seawall exposed to prevailing monsoon winds and swells (#1, Supplementary Figure S1), whereas the warm shoreline comprised a north-east-facing sheltered seawall (#2, Supplementary Figure S1). To assess the potential range of temperature conditions experienced in snail microhabitats on either shoreline, temperature-loggers (see details in Monaco et al., 2017) were deployed on rocks under direct solar exposure (a 45° angled surface), or in total shade under the rocks. Loggers were set to record temperatures every 30 min.

### Snail Collection and Laboratory Treatments

Snails (7–9 mm) were collected from both shorelines within the proximity of the temperature loggers, while awash and feeding. In the laboratory they were rinsed in freshly-collected seawater to rehydrate the snails before starting the acclimation treatments. Prior to field-fresh thermal tolerance determinations, snails were exposed to blown air (30°C for 20 min; Memmert UFE 500, Schwabach, Germany) to inactivate them, dry shells and induce withdrawal into the shell (Marshall et al., 2018). Field-fresh tolerance experiments were carried out within 12 h of collection of the snails.

**FIGURE 2:**
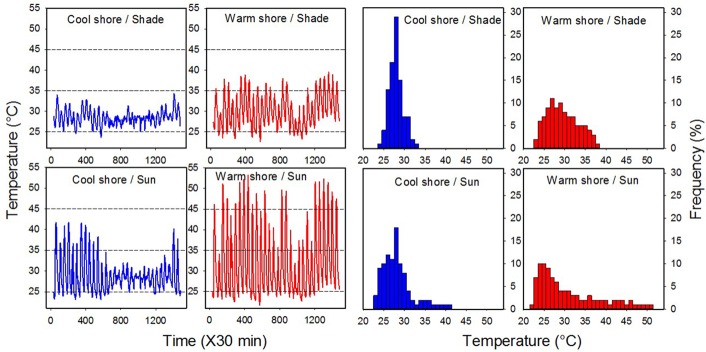
Daily field temperature regimes and thermal frequencies for the four habitats (cool shore, CS, sun and shaded, and warm shore, WS, sun and shaded). All data were logged using DS1923-F5# Hygrochron I-buttons over 30 days. Temperatures were recorded between 21 June and 22 July 2018 at the sites where snails were collected (see Supplementary Figure S1).

Four (4) primary laboratory temperature acclimation treatments were conducted on both warm-shore (WS, warm-acclimatized) and cool-shore (CS, cool-acclimatized) snails. The first set of experiments involved using a programmable Memmert Peltier-cooled (IPP400) incubator to cool-acclimate (CA) one group of randomly selected snails in air at 22–23°C, which approximates the coolest thermal regimes naturally experienced by tropical populations of *E. malacccana* (Marshall et al., 2010; Monaco et al., 2017; Figure 2 and Supplementary Figure S2). A second group was warm-acclimated (WA) to a daily 25–45°C thermal cycle, holding the temperature at 45°C for 4 h (midday) and at 25°C for 12 h (night-time), to mimic hot daily field conditions (Figure 2 and Supplementary Figure S2). Notably, whereas sun-exposure associates with high daily temperature fluctuations, cool-shaded conditions are relatively stable. These acclimations proceeded for 10 d, with snails, unfed and resting, immersed each day in flowing seawater for 5 min, to simulate tidal wetting, maintain full hydration and prevent deep aestivation (Marshall and McQuaid, 2011). Another set of experiments, which tested whether complete acclimation occurred during the above thermal treatments, involved 10 d more severe cooling [near-constant 20°C; extra-cool acclimation (ECA)] and warming (25–50°C cycle, 2 h at 50°C; extra-warm acclimation, EWA; Supplementary Figure S2). We further tested whether full acclimation occurred within a 10 d period, by assessing the effect of WA for 20 d.

### Heat Ramping and LT_50_ Determination

We assayed the effect of acute heating on mortality using the median lethal temperature (LT_50_; Marshall and McQuaid, 2011; Marshall et al., 2011, 2015, 2018). Acute overheating, rather than chronic temperature conditions that involve energetics, is the most likely cause of thermal stress related mortality in high-shore animals (Williams and Morritt, 1995; Marshall and McQuaid, 2011; Marshall et al., 2011). The critical thermal maximum (CT_max_), the temperature at which neuromuscular co-ordination fails, which is commonly used in ectotherm experiments, is unsuitable for determining gastropod lethality. This is because inactivity in high-shore littorinid snails relates to desiccation-risk-avoidance, and snails with shells glued to rock surfaces withstand temperatures well above those limiting foot physiological performance (Marshall and McQuaid, 2011; Marshall et al., 2013, 2015; Monaco et al., 2017). Much controversy surrounds the effects of ramping on acute heat tolerance determination (Terblanche et al., 2011). We selected a ramp rate of 0.25°C.min^-1^ or slightly slower, appropriate to the heating experienced in the field (Figure 2; Marshall et al., 2011). Prior to determining heat tolerance of acclimated snails, individuals were rehydrated and their shells dried as in the case of the field-fresh snails.

To determine heat tolerance, acclimated snails were placed in dry 50 ml glass tubes in a programmable bath (Grant TXF200, Cambridge, United Kingdom) and equilibrated at 30°C for 10 min, before being heated at 0.25°C.min^-1^. To maintain water bath temperature stability during the LT_50_ experiment, the heating rate was slowed to 0.12°C.min^-1^ between 50 and 60°C. Naturally, the apparent lethal temperature will be affected by time at different temperatures. Temperatures inside the test tubes were recorded every minute using calibrated K-type thermocouples connected to a TC-08 interface and Picolog software (Pico Technology, Cambridge, United Kingdom). Lethality (LT_50_) was determined for groups of 10 snails that were randomly removed from the water bath at 1°C intervals between 55 and 60°C, and allowed to recover at 28°C in wetted Petri dishes. Snails that emerged from their shells, extended their foot, and remained attached to the surface after 12 h were scored as alive. Alive but unattached snails, which are ecologically non-functional and vulnerable, were scored dead. All experiments were repeated either three or six times based on logistic constraints, with numbers of individuals limited for conservation purposes. One thousand nine hundred individual snails were used in the experiments. The study was approved by the Faculty of Science Ethics Committee, Universiti Brunei Darussalam. The effect of acclimation on LT_50_ was statistically compared between paired treatments using Generalized Linear Models (GLZM) for a binomial distribution, with a logit-link function (Statistica v12, StatSoft, New York, United States). Actual values of LT_50_ were computed from three parameter logistic regressions, which were plotted using Sigmaplot v14 (Systat Software, Inc., New York, United States).

## Results

### Field Temperature Conditions

The daily temperature conditions and thermal frequencies for four habitats, the coolest in the shade and the hottest in the sun, are shown for each shoreline in Figure 2. There was little difference between the habitats in average temperature for this period (28.5–30.1°C; Table 1). Likewise, absolute and mean daily minimum temperatures were largely invariable among the habitats (21.7–23.7 and 24.2–26.0°C, respectively). However, the maxima differed greatly (34.3–53.3 and 30.6–45.8°C, respectively), being markedly elevated in the sun-exposed habitats. Notably, in the three cooler habitats, daily temperatures did not rise above 45°C, the temperature around which a heat shock response (HSR) is induced (Marshall et al., 2011; Han et al., 2019), whereas peak temperatures in the warmest habitat surpassed 45°C on 18 (of the 30) days (Figure 2). Daily temperature variation (ΔT) was greatest in the warmest habitat (sun-exposed, WS; 21.6°C) and lowest in the coolest habitat (shade, CS; 4.6°C; Table 1); the warmest habitat exhibited the highest maximum and the lowest minimum temperatures (Figure 2 and Table 1). The stark difference in temperatures between the shores is highlighted by similar measures (means, maxs, mins, and ΔT) for the sun-exposed, CS and the shaded, WS habitats (Figure 2 and Table 1). Although several factors influence long-term temperature variations in these tropical habitats, including seasonal and El Nino effects, our recordings for a narrow timeframe are representative of relative daily thermal regime differences between the habitats and shores.

**Table 1:** Field temperatures in the four habitats, logged every 30 min for 30 days using DS1923-F Hygrochron I-buttons (see Figure 2).

	Average	Max	Min	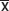 daily max	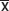 daily min	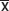 daily ΔT
Cool shore (CS) (sun)	28.95	41.69	23.13	34.91	24.98	9.93
Cool shore (shade)	28.49	34.31	23.69	30.61	26.03	4.58
Warm shore (WS) (sun)	32.06	53.25	21.69	45.79	24.16	21.63
Warm shore (shade)	30.07	39.50	22.69	35.42	25.59	9.82

### Heat Tolerance Plasticity

**FIGURE 3:**
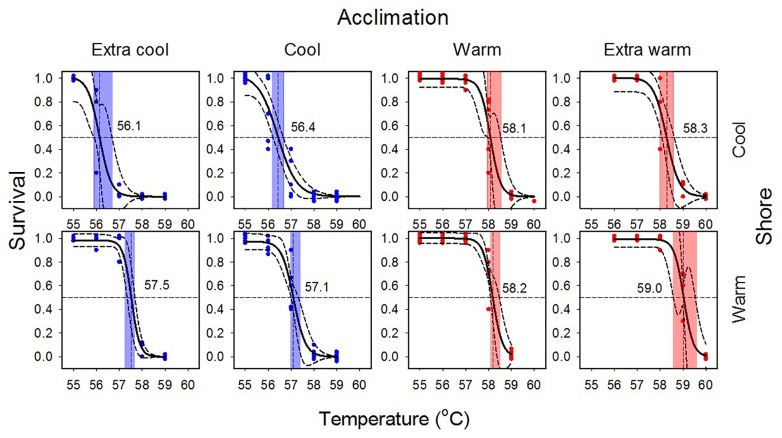
Survival curves for laboratory-acclimated *Echinolittorina malaccana* snails from the cool (CS) and the warm shore (WS). Solid black logistic regressions are based on the combined data for each trial. Dashed lines associated with regressions represent 95% CIs. Colored symbols refer to individual trials for cool and warm acclimation (CA and WA, 5 trials) and extra cool and extra warm acclimation (ECA and EWA, 3 trials) using 50 snails per trial. Vertical lines indicate LT_50_ values and their associated colored bands, their 95% CIs.

**FIGURE 4:**
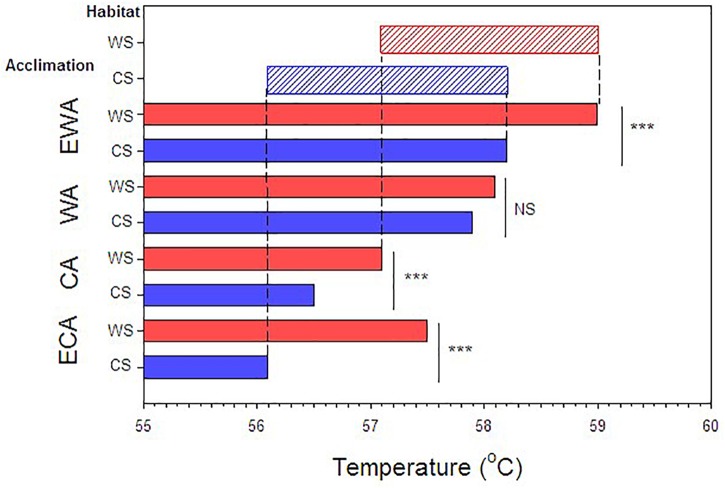
Compilation of results showing the LT_50_ values for the various acclimation and acclimatization (habitat) conditions. Red bars indicate warm shore acclimatization (WS) and blue bars, cool shore acclimatization (CS). ECA, CA, WA, EWA refer to extra-cool, cool, warm and extra-warm acclimation, respectively. Asterisk indicates *P* < 0.01 and NS, non-significant difference. Lightly shaded upper bars indicate acclimation capacities and ranges for warm shore (red) and cool shore (blue) acclimatized snails.

Substantial heat tolerance plasticity was observed in *E. malaccana* snails. The overall magnitude of their LT_50_ adjustment was 2.9°C, when accounting for all laboratory-acclimated and field-acclimatized conditions (mean LT_50_ ranged from 56.1 to 59.0; Figures 3, 4 and Table 2). Whereas snails from either shore exhibited similar magnitudes in reversible acclimation (∼2°C), the thermal bands over which acclimatory adjustments were made differed between the shores. The acclimation band for WS snails was shifted to a hotter temperature range compared to that for CS snails (Figure 4).

**Table 2:** Statistical information comparing effects of field acclimatization (shoreline) and laboratory acclimation temperature conditions on the LT_50_ of *Echinolittorina malaccana*.

Shoreline	Acclimation	LT_50_	95% CI (n)	Wald stat.	*P*<
Cool (CS)	Cool (CA)	56.4	56.2–56.7 (5)		
Warm (WS)	Cool	57.1	57.0–57.3 (5)	30.91	**0.001**
Cool	Warm (WA)	58.1	58.0–58.5 (5)		
Warm	Warm	58.2	58.1–58.5 (5)	2.33	0.127
Cool	Extra cool (ECA)	56.1	55.9–56.7 (3)		
Warm	Extra cool	57.5	57.3–57.7 (3)	31.36	**0.001**
Cool	Extra warm (EWA)	58.3	58.1–58.6 (3)		
Warm	Extra warm	59.0	58.6–59.5 (3)	16.72	**0.001**
Cool	Cool				
Cool	Extra cool			1.83	0.177
Cool	Warm				
Cool	Extra warm			3.39	0.065
Warm	Cool				
Warm	Extra cool			10.15	**0.001**
Warm	Warm				
Warm	Extra warm			22.01	**0.001**
Cool	Field	57.0	56.1–57.8 (3)		
Warm	Field	57.7	57.4–57.9 (3)	23.19	**0.001**

*LT_50_s were computed from logistic regressions (SigmaPlot Ver. 14; Figure 3) and differences between pairs of conditions were assessed from logit-linked binomial regressions (Generalized Linear Model, Statistica Ver. 13.3). Significant differences are indicated in bold.*

Cool acclimation (CA) significantly lowered the mean LT_50_ of CS snails below that of WS snails (*p* < 0.001; Table 2). Because further cooling (ECA) of CS snails did not reduce the LT_50_ further, we consider the mean hard lower limit to heat tolerance of this snail population to be 56.1°C (*p* = 0.177; Table 2). Similarly, the hard lower limit for WS snails is apparently 57.1°C (Figure 4 and Table 2). Warm acclimation (WA) markedly raised the mean LT_50_ of CS snails (58.1°C), but no further increase in heat tolerance was observed for this population with further warming (EWA, extra-warm acclimation; *p* = 0.065; Table 2), suggesting that their hard upper heat tolerance boundary is reached under WA (Figure 4). There was also no significant difference between the two shoreline populations under WA (*p* = 0.127; Table 2). However, WA apparently did not result in complete thermal acclimation of the WS snails, as their heat tolerance rose following extra-warm temperature acclimation (EWA, LT_50_ = 59.0°C; *p* < 0.001, Table 2).

Mean field-fresh LT_50_ was greater in WS snails (57.7°C) compared to CS snails (57.0°C; *p* < 0.001; Table 2), reflecting the different recent thermal histories experienced on the different shorelines. There was no difference in LT_50_ between the 10 d and 20 d WA treatment for warm shore snails, indicating that 10 d is sufficiently long to produce complete compensation (*p* = 0.295). When the cool and warm habitat data were combined (CS and WS), and compared with the data for WS snails, the combined data set showed significantly lower LT_50_ values for ECA, CA, and EWA (*p* < 0.007; Table 3), but not for the WA laboratory treatments (*p* = 0.369; Table 3). While it was not investigated in the present study, individual variation can be as important an endpoint as the mean value in any study investigating temperature responses.

**Table 3:** Statistical information comparing effects of warm-shore (WS) and combined-shore (warm and cool) on the LT_50_ of *Echinolittorina malaccana* for the various acclimation treatments.

Shoreline	Acclimation	LT_50_	95% CI (n)	Wald stat.	*P*<
Warm	Cool				
Combined	Cool	56.7	56.5–56.9 (10)	11.85	**0.001**
Warm	Warm				
Combined	Warm	58.1	58.0–58.4 (10)	0.81	0.369
Warm	Extra cool				
Combined	Extra cool	56.9	56.4–57.3 (6)	18.71	**0.001**
Warm	Extra warm				
Combined	Extra warm	58.7	58.4–58.9 (6)	7.34	**0.007**

*LT_50_ values for WS are given in Table 2. Significant differences are indicated in bold.*

## Discussion

Ectotherms vary widely in ability to adjust physiological performances and tolerances in response to lifetime changes in environmental temperature (Angilletta, 2009). Contrary to the prediction of mainstream theory that thermal acclimation is relatively constrained in tropical and high-intertidal animals (Stillman and Somero, 2000; Stillman, 2002; Somero, 2005, 2010; Gunderson and Stillman, 2015; Rohr et al., 2018), we found substantial plasticity in the lethal thermal limit (LT_50_) of *Echinolittorina* snails (see also, Marshall et al., 2018). However, we additionally, show that heat tolerance plasticity of these snails comprises both a reversible and a non-reversible component. Reversible plasticity was induced by laboratory acclimation and non-reversible plasticity was shown by differences in the thermal bands for lethal temperature (LT_50_) acclimation between populations from thermally-different shorelines (see Figure 4). Snails from the warmer shore were found to shift the band for thermal acclimation to a higher range of temperatures compared to the CS snails. These different forms of plasticity align with different spatiotemporal scales of the environmental temperature variation. Reversible plasticity facilitates thermal tolerance adjustments in response to daily or tidal habitat temperature variation, whereas non-reversibility canalizes or fixes the thermal tolerance to shoreline-specific temperature conditions throughout the individual’s lifetime.

### Reversible and Non-reversible Plasticity

Reversible plasticity enables individual organisms to adjust performances and tolerances in response to cycling temperatures for timeframes from seasons to days. In comparative studies, laboratory acclimation is typically performed to eliminate the effect of recent field temperatures on the physiological performance of individuals. Our observed persistence of phenotypic differences after laboratory acclimation in populations from thermally-different shorelines (Figure 3 and Table 2) suggests an effect of heritable differences between the populations or non-reversible plasticity. Because the snail larvae settling on either shoreline were randomly drawn from the same planktotrophic pool, comprising individuals derived from multiple different parents, we disregard local adaptation or other inherited effects as the cause of the observed population difference (Schmidt and Rand, 1999; Williams and Reid, 2004; Kelly et al., 2011; Foo and Byrne, 2016). Non-reversible plasticity has been described as transgenerational, such as non-genetic heat-hardening transferred to the embryo from the parent, or as developmental, such as post-embryonic heat-hardening (Angilletta, 2009; Donelson et al., 2011, 2012; Reusch, 2014). Using the same argument as above for random shoreline recruitment of larvae, the shoreline (population) differences in thermal plasticity can also not be explained by a transgenerational response to heat exposure (Williams and Reid, 2004). Our findings therefore suggest that non-reversible heat tolerance is most likely founded after larval snails have settled on the shore; thus, shoreline differences are best described as developmental plasticity (Angilletta, 2009). Because the crawling snails occupy meter-size habitats, cross-shore migrations can be discounted. Notably, the apparent developmental plasticity should be reinforced by the shoreline-specific temperature conditions during the lifetime of each snail, from the crawling juvenile to the adult (Angilletta, 2009; see Slotsbo et al., 2016 on reversibility of developmental plasticity).

### Environmental Temperature and Molecular Underpinnings of Plasticity

Molecular processes underlying thermal plasticity are cued by different components of the thermal regime (mean, maxima, and minima) for different durations of thermal cycling (daily or seasonal). Whereas seasonal acclimatization, regulated by isozyme expression (Hochachka and Somero, 2002), is typically cued by mean temperature change (Angilletta, 2009), acute daily heating of rocky-shores elicits thermal plasticity through a HSR, triggered by peak (maximum) temperatures (Hofmann and Somero, 1996; Feder and Hofmann, 1999; Tomanek and Somero, 1999; Somero et al., 2017). Although HSRs are complex, involving upregulation of multiple heat shock proteins (Hsps), much information can be gleaned from individual gene *thermal expression profiles* (relative levels and thermal ranges of expression) and *thermal thresholds* (Tomanek and Somero, 1999; Wang et al., 2017; Han et al., 2019). Previous studies on *E. malaccana* showed that *hsp70* expression profiles differ among geographically-separated populations (Wang et al., 2017; Han et al., 2019), such that reduced expression correlates with populations from cooler locations and *vice versa* (Wang et al., 2017; Han et al., 2019). The same mechanism in a spatially scaled-down form could underlie the heat tolerance differences observed between our cool and warm shore populations. Whereas these findings imply plasticity of *hsp* expression profiles, studies for diverse populations of *E. malacanna* suggest that the HSR thermal threshold for this species may otherwise be fixed at around 45°C (Marshall et al., 2011; Han et al., 2019; see also Hoffmann et al., 2003). Interestingly, whereas our cool and warm shore snails adjusted heat tolerance to the same level when acclimated to a daily maximum of 45°C, only the WS snails that commonly experience temperatures above 45°C responded positively to the extra-warm acclimation treatment (EWA; for which the daily thermal peak was 50°C; Figure 3 and Table 2).

Whereas daily maximum temperatures varied greatly across the snail habitats, mean field temperatures (which typically drive seasonal acclimatization) were largely similar across habitats (sunned and shaded) and shorelines (cooler and warmer; Table 1). Clearly, mean temperatures contribute insignificantly to heat tolerance plasticity selection in tropical *Echinolittorina* snails. Likewise, minimum field temperatures were largely invariable across the habitats and shores, which excludes these temperatures as potential determinants of the lower boundary for heat tolerance plasticity. The limit to this boundary, assessed during CA for both shores, possibly relates to the loss of heat-hardening, a mechanism which should be beneficial by eliminating costs associated with the upregulating and functioning of Hsps (Angilletta, 2009).

### Benefits of Dual Heat Tolerance Plasticity

Plastic responses involving Hsps incur significant energetic and fitness costs, a topic extensively critiqued in the framework of the *beneficial acclimation hypothesis* (Leroi et al., 1994; Huey et al., 1999; Wilson and Franklin, 2002; Woods and Harrison, 2002; Deere and Chown, 2006). In their high-shore habitat, *Echinolittorina* snails face severe energy intake restrictions due to limited food availability and limited time in which to feed. Consequently, they have evolved multifaceted behavioral and physiological mechanisms to conserve energy resources (Marshall et al., 2011, 2013; Marshall and Chua, 2012). Among the most impressive energy-conserving mechanism is deep temperature-independent metabolic rate depression (10% of resting metabolism; Marshall et al., 2011; Verberk et al., 2016). In view of their energetic constraints, advantage should be gained by minimizing the use of costly physiological processes, including HSRs.

Non-reversible plasticity should be energetically beneficial by ensuring that the range for reversible heat tolerance plasticity matches the habitat temperatures of a particular shore. Such matching should prevent snails on warmer shores from experiencing excessive HSR induction and *hsp* overexpression (Feder and Hofmann, 1999; Sørensen et al., 2003). Non-reversible heat tolerance plasticity should enable reversible acclimatization to occur in similar ways and at similar costs on thermally-different shores under the same regional change in ambient air temperature. Furthermore, this plasticity should yield species-level benefits by enabling colonization of a broader range of shores (warmer and cooler shores) along a coastline. Importantly, these findings accounting for non-reversibility supersede an earlier suggestion that *E. malaccana* is unlikely to benefit from reversible plasticity (Marshall et al., 2018).

### Taxonomic Generalization and Habitat Heterogeneity

Our understanding of thermal acclimation of rocky-intertidal animals in the context of contemporary theory (Angilletta, 2009) is encompassed by the influential work of Somero and Stillman (see Stillman, 2002; Somero, 2005, 2010). This work suggesting a reduced acclimation capacity in higher-shore species compared to their lower-shore congeners, arises from generalization of data for porcelain crabs (Stillman, 2002; Somero, 2005, 2010). These crabs, however, behaviourally thermoregulate by sheltering in the shade under rocks during air emersion, limiting their exposure to the full spectrum of the shoreline’s thermal heterogeneity. The substantial heat tolerance plasticity observed in high-shore *Echinolittorina* snails contradicts this theory, and we suggest that the difference between the crab and snail responses relates to the snails using a broader spectrum of the shoreline thermal heterogeneity. They experience relatively great habitat temperature variation through the behavior of settling when air-exposed in thermally-divergent microhabitats, including those under direct solar heating (Marshall et al., 2010, 2013). This habitat temperature variation persists despite the phenomenal variety of morphological and behavioral thermoregulatory attributes of littorinid snails (Miller and Denny, 2011; Marshall and Chua, 2012; Marshall et al., 2013; Ng et al., 2017), primarily because the most effective thermoregulatory behavior, shade-seeking, can be undermined by desiccation risk avoidance behavior (Monaco et al., 2017).

An arising question is whether capacity for non-reversible plasticity (the shoreline effect) is restricted to high-shore species or whether it is independent of vertical distribution on the shore. Because lower-shore habitats are strongly stabilized by the seawater temperature, and because the seawater temperature is largely invariable across nearby shorelines, the shoreline effect should be lessened in the lower-shore. In addition to suggesting that seawater temperature stability is likely to restrict thermal acclimation selection in lower-shore tropical species, we propose as a testable hypothesis that non-reversible plasticity should also be more constrained in lower-shore compared to higher-shore species.

### Methodological Implications

Acclimation capacity (the degree of change in a trait following cool or warm laboratory acclimation) is becoming a key measure of the vulnerability of ectothermic animals to future warming (Gunderson and Stillman, 2015; Rohr et al., 2018). An earlier study revealed that laboratory experiments alone (without field-referencing) may underestimate this vulnerability in animals living in near-completely acclimatized states, which are unable to improve heat hardening with further warming (Marshall et al., 2018). The present study adds to this caveat by showing that inaccuracies in determining heat-tolerance acclimation capacity can potentially arise from not accounting for shoreline-specific temperature differences. Whereas the capacity for reversible acclimation was similar for each shoreline (∼2°C), this became greater when the data for the shores were combined (∼2.9°C; Table 3 and Figure 4). This highlights the importance, when assessing acclimation capacities, of prior knowledge of the habitat thermal heterogeneity of experimental animals.

Emerging from this study is a second methodological issue relating to diel cycling of the laboratory acclimation temperature. This is not only important considering that such cycling occurs naturally, but also in terms of initiating an acclimatory HSR. If the primary heat-hardening response requires that exposure temperature surpasses an HSR induction threshold, then acclimation temperatures that are stable or fluctuate below this threshold will conceivably not yield an acclimatory response, leading to erroneous conclusions. This further highlights the need to determine the HSR threshold temperature prior to developing acclimation protocols in marine intertidal animal studies.

## Conclusion

Our study adds an important dimension to the existing theory proposing that thermal tolerance plasticity is relatively constrained in tropical ectotherms. In particular, it reveals that this plasticity can be complex in thermally-heterogeneous tropical marine intertidal ecosystems. Whereas the contribution of marine intertidal circumstances to a body of theory for thermal plasticity developed largely from terrestrial and subtidal animals might be questionable, our findings nonetheless caution against the indiscriminate use of this theory when interpreting intertidal data. We further show that without critical consideration of the thermal heterogeneity at the scale of the organism and how this heterogeneity may drive different forms of thermal tolerance plasticity, investigations risk generating misleading conclusions.

## Author Contributions

AB and DM developed the hypothesis and prepared the manuscript. AB and NM carried out the experimental laboratory work. DM undertook the field recordings. NM reviewed the manuscript.

## Conflict of Interest Statement

The authors declare that the research was conducted in the absence of any commercial or financial relationships that could be construed as a potential conflict of interest.
