# Supplementary material for: Non-reversible and Reversible Heat Tolerance Plasticity in Tropical Intertidal Animals: Responding to Habitat Temperature Heterogeneity
Source: Front Physiol. 2019 Jan 14;9:1909. doi: 10.3389/fphys.2018.01909 (PMC6339911; doi:10.3389/fphys.2018.01909)
Supplement: Supplementary file 1 [file Image_1.pdf]

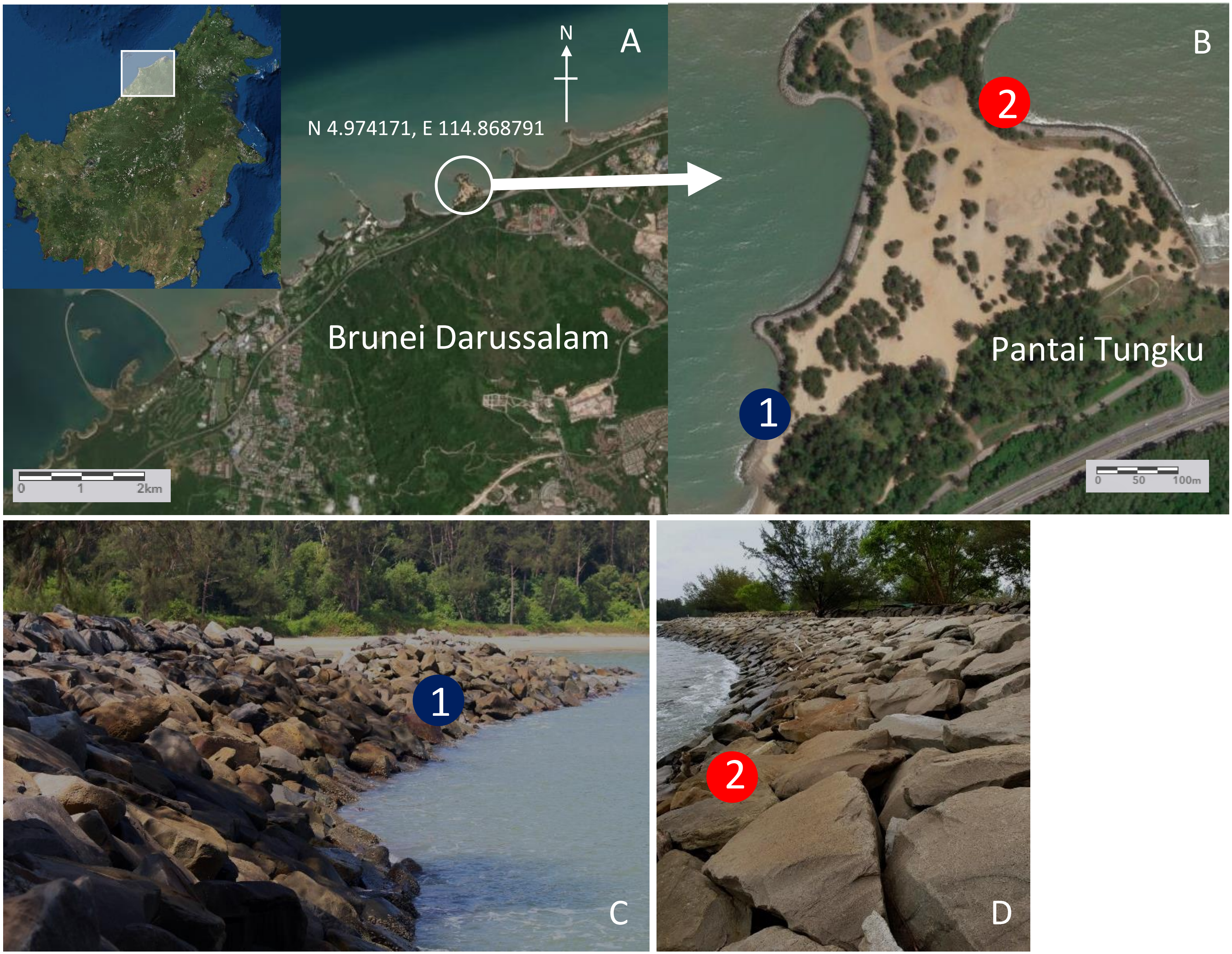
A
N
N 4.974171, E 114.868791
Brunei Darussalam
0 1 2km
B
2
Pantai Tungku
1
0 50 100m
1
C
2
D

Supplementary Figure. 1. Satellite images of the locations of the shores, including map of Borneo (A and B). C and D are images of the seawalls and habitats where *Echinolittorina malaccana* were collected and I-buttons deployed. Site #1 is facing north westwards into the prevailing winds and swells. Site #2 is facing north eastwards and is sheltered from the prevailing winds and swells.
