# Supplementary material for: Non-reversible and Reversible Heat Tolerance Plasticity in Tropical Intertidal Animals: Responding to Habitat Temperature Heterogeneity
Source: Front Physiol. 2019 Jan 14;9:1909. doi: 10.3389/fphys.2018.01909 (PMC6339911; doi:10.3389/fphys.2018.01909)
Supplement: Supplementary file 2 [file Image_2.pdf]

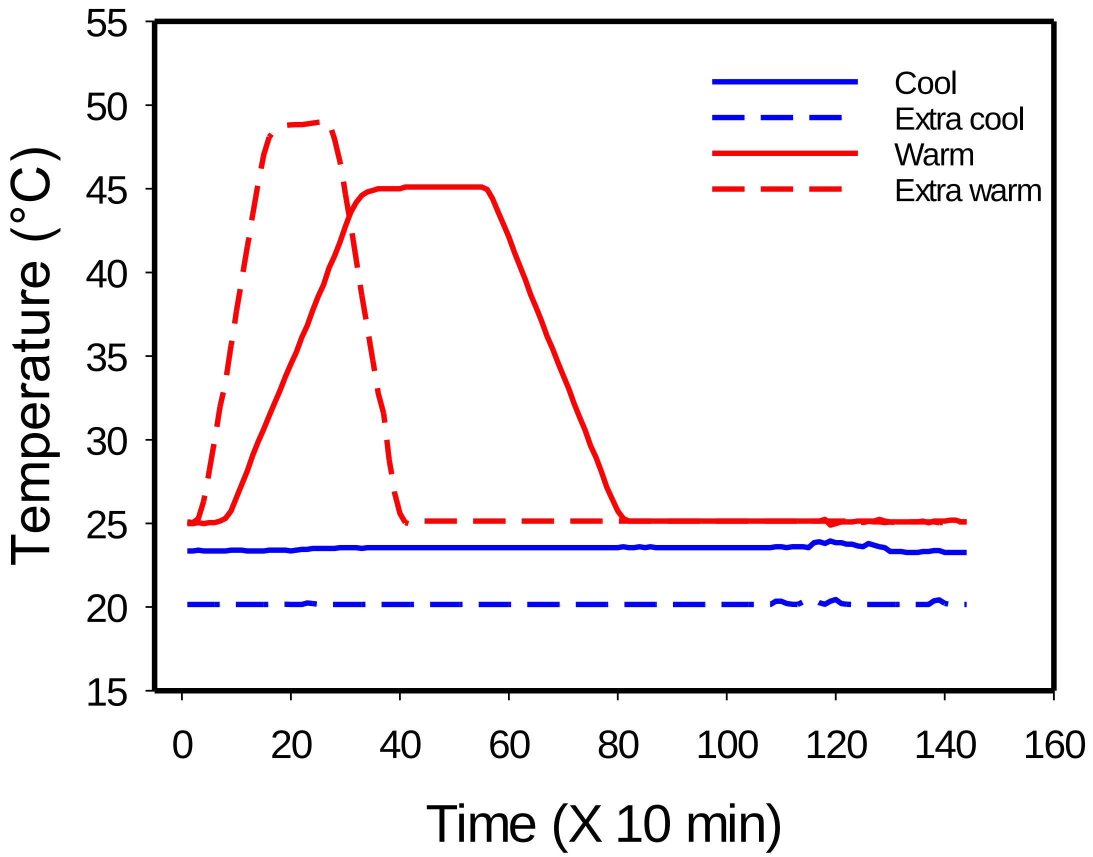

Supplementary Figure. 2. The laboratory acclimation protocol showing the temperature variation over 24 h for the four treatments, extra cool (ECA), cool (CA), warm (WA) and extra warm (EWA). Snails were acclimated in an incubator (Memmert UFE 500, Schwabach, Germany) and the temperature was recorded using DS1923-F Hydrochron I-buttons for 10 days.
